# Winter Wisdom

**Published:** 1873-02

**Authors:** 


### WINTER WISDOM.

In going from a warm room into the cold of out-doors, especially if there is a raw, damp wind, wrap up, button up, and draw on the gloves before opening the door, then keep the mouth resolutely closed so as to cause the air to pass through the nose and the long circuit through the head, thus warming it before it reaches the lungs. Public speakers are especially enjoined to take this precaution; many a valuable life has been lost from inattention to this point.

Never stand at the crack of a door in winter for "last words;" in cold weather it is better far to send a servant to the door with your guest.

Never allow the family to come into the breakfast-room of a winter morning until it is heated to sixty-five degrees, especially if the children have to wash and dress in a room without fire. No one ought ever to eat in a chill. If, in sitting down to a meal, you are cold or very tired, take some hot soup or hot drink of some kind, and then wait a few minutes, or eat very slowly at the beginning of the meal.

In coming home and not expecting to go out again for the day, draw off the shoes and stockings, hold the naked feet to a blazing fire, rubbing them well with the hands until most thoroughly warm and dry, then draw on a pair of dry socks or stockings and put the feet into soft, warm slippers: there will be a comfort in this which will richly repay the trouble of the procedure.

If you purpose remaining in a room half an hour as a caller, do not lay aside the overcoat or shoes, even if the atmosphere of the apartment seems oppressive; it is always cooler than it seems to be, and it is better to go out of doors a little too warm than on the verge of a chill.

If you have to walk and ride on a cold day, do the riding first and then warm yourself up by a walk, for if you get warm in walking and sit still in a cold vehicle, with the chances of some one opening a window, you will inevitably cool off too rapidly, endangering a chill, a bad cold or an attack of pneumonia or inflammation of the lungs.

Never open a window on entering a vehicle; it is an impertinence of which no gentleman or lady could possibly be guilty; if the apartment is too warm, or too confined for you, those who have been sitting still are quite cool enough already, and there is no reason why you should discompose half a dozen or more persons for your own accommodation. Under the circumstances it is the essence of selfishness to ask permission to open a window, for cultivated persons would risk a personal injury rather than seem boorish by a refusal.

If it is cold enough to freeze outside, it is safer to keep the outer windows of the chamber closed, and to keep an inner door and fireplace open.

